# Ultrasound Guided Intra-Articular Injection of Triptolide-loaded Solid Lipid Nanoparticle for Treatment of Antigen-Induced Arthritis in Rabbits

**DOI:** 10.3389/fphar.2022.824015

**Published:** 2022-02-17

**Authors:** Shilin Li, Liyang Su, Guorong Lv, Weiwen Luo, Yishan Kang

**Affiliations:** ^1^ Department of Ultrasonography, The Second Affiliated Hospital of Fujian Medical University, Quanzhou, China; ^2^ Department of Ultrasonography, Quanzhou First Hospital, Fujian Medical University, Quanzhou, China; ^3^ Quanzhou Medical College, Clinical Medicine Quanzhou, Quanzhou, China

**Keywords:** intra-articular injection of triptolide-loaded solid lipid nanoparticle triptolide, nano, interventional ultrasound, intra-articular injection, rheumatoid arthritis

## Abstract

**Objective:** To evaluate the efficacy of ultrasound-guided intra-articular injection of triptolide-loaded solid lipid nanoparticle (TP-SLN) for treatment of antigen-induced arthritis (AIA) in rabbits.

**Material and Methods:** Knee joints of 33 New Zealand rabbits with AIA were injected intra-articularly with triptolide (TP: n = 7), TP-SLN (n = 7), betamethasone (BS: n = 7) and dimethyl sulfoxide (DMSO: n = 6). The remaining six rabbits were untreated as the control group. The injection therapy in intervention groups was initiated 1 week after the last immunization in order to avoid irreversible joint damage in the later induction. The ultrasonic scores of the joints were assessed based on synovitis, synovial blood flow and bone erosion. Meanwhile, the correlations of ultrasonic scores and pathological scores were determined. The efficacy and side effects of each group were determined by combining ultrasonic scores, pathological scores, behavior, appetite, weight, joint diameter, skin temperature and biochemical examination.

**Results:** 1) Compared with the control group, the diameters of knee joints of the TP, TP-SLN and BS groups began to reduce 1 week after intra-articular injection (*p* < 0.01). 2) With the exception of the DMSO group, the interventions were effective in treating synovitis compared with the control group, with TP-SLN and BS being the best. The ultrasonic and pathological scores in synovitis of the TP group were lower than that of model group (Z = -2.726 and -2.530, *p* < 0.05). The ultrasonic scores differed significantly between BS group and TP-SLN group (Z = −2.17 and -2.360, respectively, *p* < 0.05) and pathological scores (Z = −2.687 and −2.082, respectively, *p* < 0.05). 3) Compared with the control group, the TP, BS and TP-SLN were all effective in treating synovial blood flow and bone erosion and there were no significant differences of ultrasonic and pathological scores among them (*p* > 0.05). The ultrasonic scores of synovial blood flow (Z = −3.033, −2.842, −3.277, *p* < 0.01) were lower than in the controls. The ultrasonic scores (Z = -2.948, -3.141, -3.210, *p* < 0.01) and pathological scores (Z = −2.216, −2.505, −2.505, *p* < 0.05) of bone erosion were also lower than in the model group.4) There were significant correlations between the ultrasonic and pathological scores of synovial inflammation and bone erosion (r = 0.832 and 0.859 respectively, *p* < 0.001).

**Conclusions:** The therapeutic effect of TP-SLN on arthritis is better than that of TP, but there is no difference between BS and TP-SLN. Therefore, TP-SLN may be used as an alternative to BS in the treatment of rheumatoid arthritis in the future. The ultrasonic and pathological scores showed significant correlation in synovitis and bone erosion. Ultrasound can provide a useful assessment of synovitis in early arthritis.

## 1 Introduction

Rheumatoid arthritis (RA) is a chronic, inflammatory polyarthritis with frequent progression to joint destruction and disability. Often, only a brief period of time elapses between the onset of joint inflammation and the development of bone erosions and cartilage injury (Firestein and McInnes, 2017). Many studies have estimated the prevalence of RA around the globe. A systematic effort, undertaken in the first decade of the century to determine the global burden of RA, estimated the global prevalence of RA to be 0.24% (95% CI, 0.23–0.25%), with no discernible change from 1990 to 2010 (Otón and Carmona, 2020).

In light of the large problem of side effects with systemic medication, intra-articular injection of drugs has been widely used for RA. This procedure can alleviate pain and improve local joint symptoms by inhibiting the inflammatory response, especially for patients with oligoarthritis. In recent years, many scholars have focused on traditional Chinese medicine, with Tripterygium wilfordii being a main focus. Triptolide (TP), extracted from the root of Tripterygium wilfordii Hook f, has been used in Chinese traditional medicine for the treatment of autoimmune diseases and inflammatory dermatoses including rheumatoid arthritis, kidney disease, autoimmune disease, systemic lupus erythematosus and dermatomyositis (Song et al., 2019). TP is highly toxic by both oral and intravenous administration. It can inhibit the mitochondrial respiratory chain and cause liver damage. Mild damage can cause fatty liver disease, and severe damage can cause irreversible damage to the liver (Fu et al., 2011). Studies have shown that organic cation transporter two is highly expressed in collagen-induced arthritis rats and can be transported into the renal parenchyma when intravenously injected with TP, resulting in kidney damage. Additionally, TP can cause diseases of the blood system, such as leukopenia, hemoglobinuria and thrombocytopenia. However, intra-articular injections can reduce these side effects (Li et al., 2020).

Our previous studies have shown that intra-articular injection of TP can reduce collagen-induced arthritis inflammation in rats, but the treatment effect is not very strong (unpublished data). The metabolism is very fast in the joint cavity, and the drugs injected directly into the joint cavity will be rapidly cleared away, meaning it is not easy for the drug to be fully absorbed and utilized. Mitragotri and Yoo (2011) found that the micro nano system can selectively adsorb on inflammatory synovial cells as the carrier of anti-RA drugs. When these drugs leave the joint cavity, they will not cause damage to other parts of the body, so they can achieve a similar effect as targeted treatment. Solid lipid nanoparticles (SLN) offer unique properties such as small size, large surface area, high drug loading and the interaction of phases at the interface, and are attractive for their potential to improve performance of pharmaceuticals (Ghasemiyeh and Mohammadi-Samani, 2018).

For this study, we generated an antigen-induced arthritis (AIA) model. In order to avoid the irreversible joint damage in the later stage, we injected drugs into the joint cavity under the guidance of ultrasound 1 week after the last induction, and then compared the efficacy and safety of each group after injecting TP-SLN, TP, BS and DMSO into the joint cavity.

## 2 Material and Methods

### 2.1 Animals

Thirty-three New Zealand rabbits, age 6 months, with a mean weight of (2.7 ± 0.2) kg, provided by Yuhuashan natural ecological agriculture test field, Lianjiang, Fujian Province (rodent license of the laboratory: SCXK (min) 2014-0001). The animals were maintained in the animal facility of Quanzhou Medical College (rodent license of the laboratory: syxk (min) 2016-0001). The rabbits were given adaptive feeding for 1 week. During the course of the experiment, the rabbits were maintained at room temperature (20 ± 1°C) and each group was caged separately and fed freely in natural light with regular cleaning and disinfection. This study was approved by the ethics committee of the Second Affiliated Hospital of Fujian Medical University (No. 20180536).

### 2.2 Reagents and Instruments

Knee joints performed using a general-purpose ultrasonic device (Hi Vision Avius; Hitachi Aloka Medical Corp., Tokyo, Japan) with a 18 MHz ultrasonic linear probe (Hitachi Aloka Medical Corp.). Purified triptolide was obtained from Beijing Bailingwei Technology Co., Ltd. (Beijing, China), BS was purchased from Mreck Sharp & Dohme AG (Basel, Switzerland). Ovalbumin and Freund’s complete adjuvant were purchased from Sigma (Missouri, United States). Precirol^®^ ATO five and Compritol 888 ATO were purchased from Sinopharm Chemical Reagent Co., Ltd (Shandong, China). GeleolTM and Cremophor RH 40 were purchased from Yousuo Chemical Technology Co., Ltd (Shandong, China). Palmitic acid was purchased from Yousuo Chemical Technology Co., Ltd (Qingdao, China). Stearic acid was purchased from Zhiyuan Chemical Reagent Co., Ltd (Tianjin, China). Sodium cholate was purchased from McLean Biochemical Technology Co., Ltd (Shanghai, China).

### 2.3 AIA Model

Animals were weekly sensitized at four to five sites on the back by subcutaneous injection of a total of 1 ml of an emulsion of equal volumes of ovalbumin with Freund’s complete adjuvant for three consecutive weeks. At the fourth week, 10 mg of ovalbumin (0.2 ml) was injected into the knee joint for the second sensitization (Kristensen et al., 2010).

**FIGURE 1:**
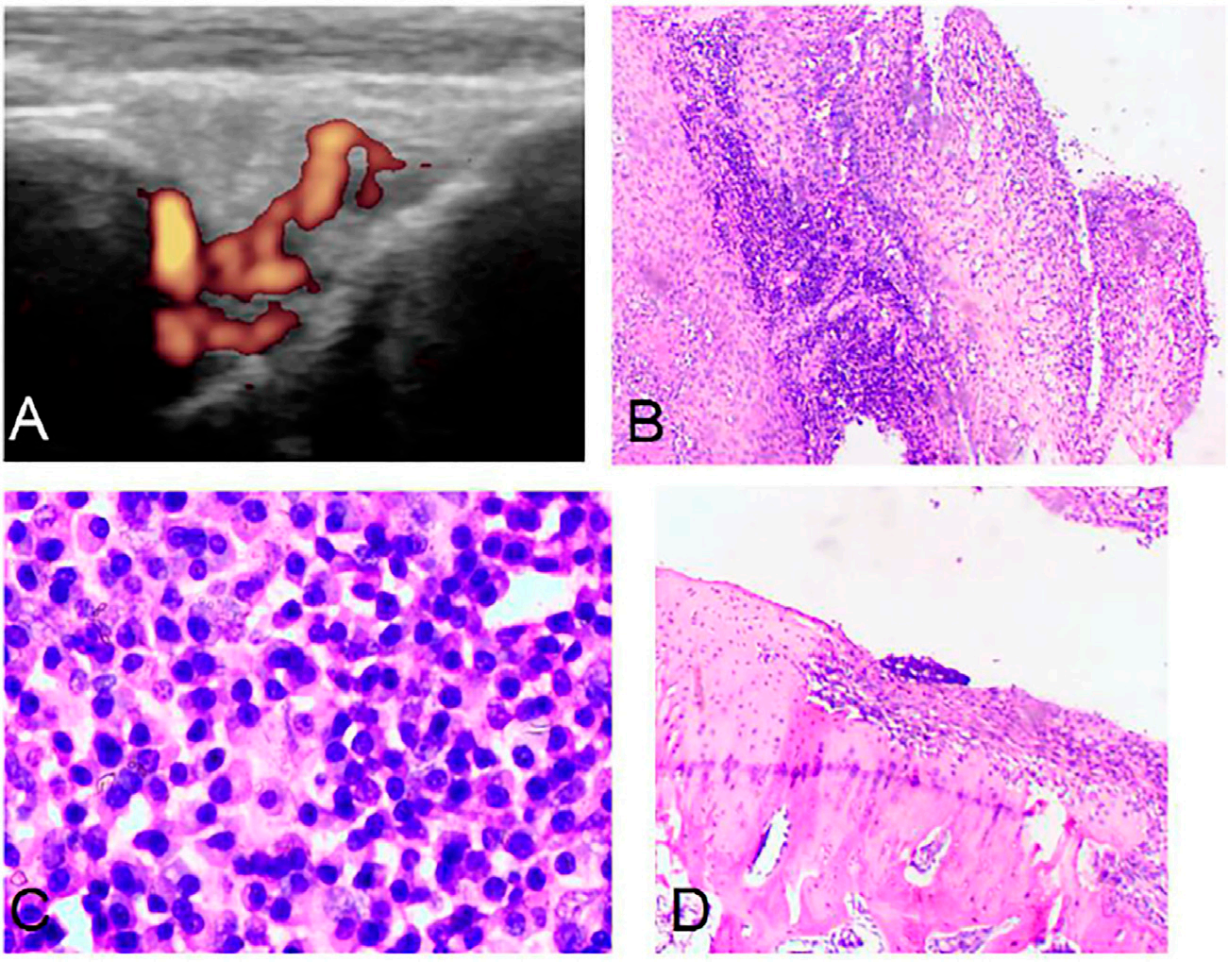
Ultrasound and pathology of AIA model. Synovitis and high blood flow signals observed by Power Doppler Ultrasound **(A)**. Synovitis and inflammatory cell infiltration can be observed by pathology **(B)** (HE, 40×). Large amount of plasma cell infiltration **(C)** (HE, 200×). Extensive bone destruction on the bone surface **(D)** (HE, 40×).

In the control group, it was found that the AIA model began to demonstrate synovium hyperplasia and synovium blood flow increase one to two weeks after the second sensitization, and bone erosion occurred four weeks later. A significant amount of synovium hyperplasia, pannus and inflammatory cell infiltration was found on the surface of the knee joint. Bone erosion was found on the pathological Section 4 weeks after the second sensitization (Figure 1).

### 2.4 Preparations of TP-SLN

TP-SLN was prepared by a microemulsion method (Ugazio et al., 2002). Briefly, TP was added to a mixture of precirol^®^ ATO 5, compritol 888 ATO, geleolTM, cremophor RH 40, palmitic acid, stearic acid and sodium cholate, and melted together in the heat collection type magnetic heating stirrer (>85°C) with magnetic stirring for 15 min. Next, ultra pure water at the same temperature was added slowly. After forming the microemulsion (light blue milky light), magnetic stirring was continued for 10 min, then the sample was quickly cooled to 4°C using ice water (the ratio of microemulsion to ice water was 1:5). The microemulsion was magnetically stirred for 10 min, and the sample was stored in the refrigerator at 4°C.

**FIGURE 2:**
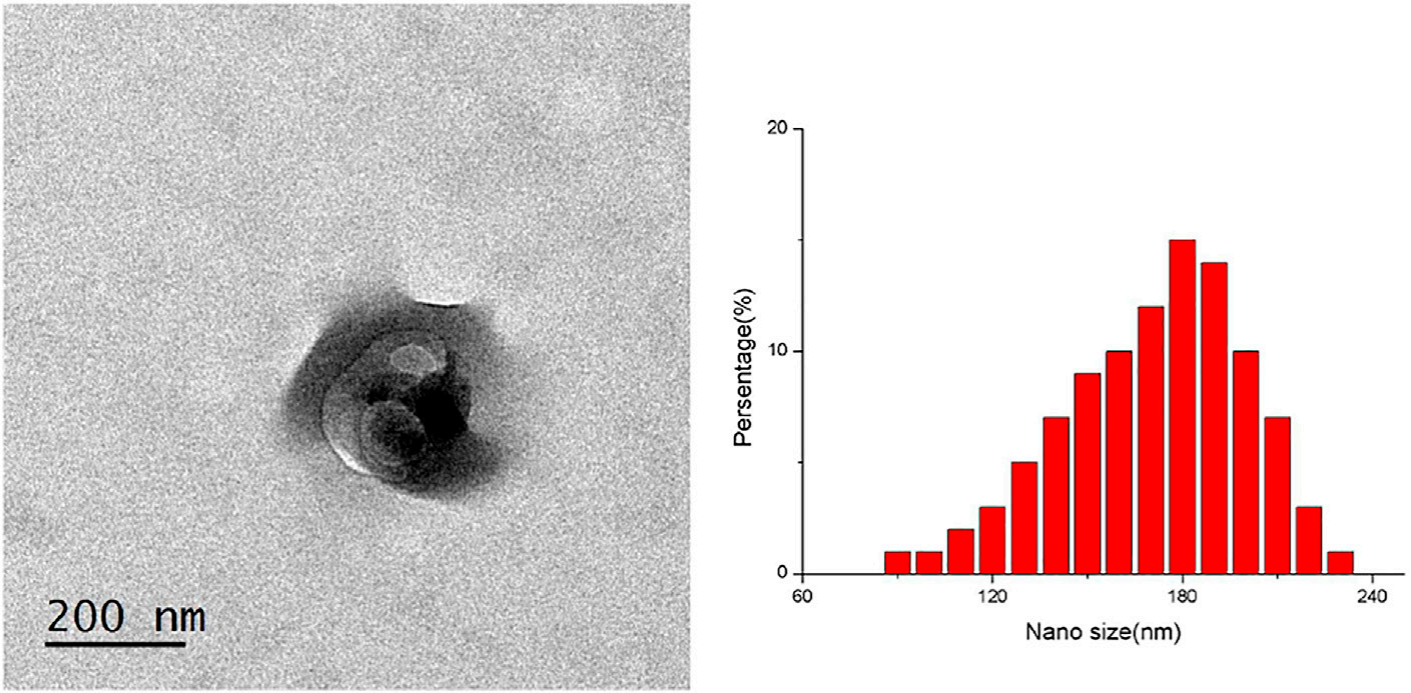
TP-SLN particles observed by TEM and zeta potential of TP-SLN.

TP-SLN did not agglutinate, and its particle size and distribution were uniform, which was confirmed with the transmission electron microscope. The particle size of TP-SLN was (181 ± 73) nm, and the zeta potential was −33 mv (Figure 2). High performance liquid chromatography (HPLC) was used to measure the entrapment efficiency and drug loading of TP-SLN.

**FIGURE 3:**
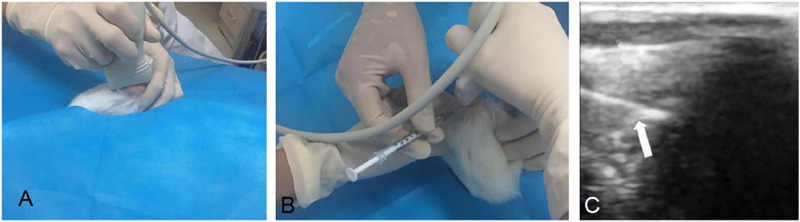
Ultrasound guided intra-articular injection. Knee joint of rabbits were scanned longitudinally with ultrasound **(A)**; transverse scan of knee joint parallel to the needle feeding direction **(B)**; ultrasound of the needle (white arrow) punctured into the articular cavity **(C)**.

### 2.5 HPLC Analysis of TP

The amount of TP penetrated into the receptor compartment was determined with a slight modifification of the reversed-phase HPLC method described previously (Xu et al., 2019).

HPLC (Agilent Technologies Inc., United States) was equipped with a Hypersil 18C column and the mobile phase consisted of methanol–water (45:55, v/v) at a flflow-rate of 2 ml/min. The retention time was 30min. Detection was accomplished using UV absorbance at 215 nm. The assay was linear (r2.0:996) in the concentration range 50–1000 ng/ml with a lowest detection limit at 18 ng/ml of TP. The percentage recoveries ranged from 93.2 to 103.7.

### 2.6 Ultrasound Guided Intra-articular Injection

Knee joints of rabbits were injected intra-articularly guided by ultrasound with 0.2 ml DMSO (n = 6), 0.2 ml BS (n = 7), 0.2 mg TP (dissolved in 0.2 ml DMSO; n = 7) and 0.2 ml TP-SLN (containing TP 0.2mg; n = 7) once a week after the second sensitization for 5 weeks (Figure 3). The control group (n = 6) remained untreated. All rabbits were sacrificed in the fifth week by intravenous air injection through the ear edge.

### 2.7 Histological Examination

Arthritic knee joints were removed post-mortem, fixed in 10% buffered formalin, and then decalcified in 10% EDTA solution for 1–2 months. The joints were then embedded in paraffin, sectioned, and stained with hematoxylin and eosin for microscopic evaluation.

### 2.8 Assessment of Treatment Effect

The ultrasonic score of synovitis was based on the semi-quantitative standard of Szkudlarek et al. (2001): score 0: no obvious thickening of synovium; score 1: mild synovitis can be seen, but it does not exceed the line of the highest point on the bone surface; score 2: the line is higher than the highest point of bone surface, but not higher than the backbone; score 3: over the line of the highest point of the bone surface and extending over one side of the shaft.

The pathological scoring was performed by an experienced pathologist according to Ellegaard et al. (2012).

The ultrasound score of synovial blood flow was based on Newman et al. (1996): score 0: no color blood flow signal; score 1: dot color blood flow signals; score 2: short linear color blood flow signal with distribution less than 1/2 of synovial area; score 3: reticular blood flow signals distributed more than 1/2 of synovial area.

Bone erosion was evaluated according to the scoring standard of Disler et al. (2000): score 0: no bone damage on the surface of bone; score 1: the surface of bone is not smooth, but there is no bone destruction; score 2: slight bone defect on bone surface; score 3: extensive bone destruction on bone surface.

### 2.9 Statistical Analysis

Data are expressed as means ± SD, and analyzed by SPSS 21.0 software. The *t*-test was used for comparison between groups. The Kappa test was used to test consistency, kappa ≥0.75 indicated good consistency. The Mann-Whitney *U* test was used to compare the grade data between the two groups, and the Kruskal Wallis test was used to compare the grade data between the multiple groups. The Spearman test was used for correlation analysis of rank data. *p* values less than 0.05 were considered statistically significant.

## 3 Results

### 3.1 Diameter of Knee Joint in Each Group

**FIGURE 4:**
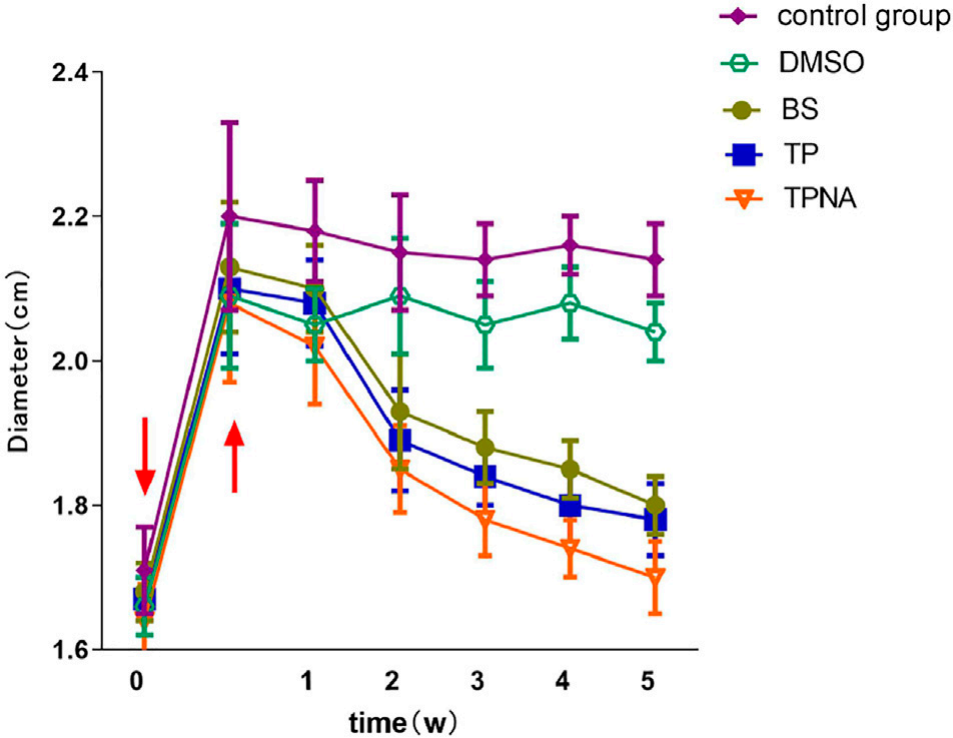
Changes of knee joint diameter in each group of experimental rabbits. Induction by injection of ovalbumin into the articular cavity (↓). After 1 day, the diameter of the knee joint increased significantly (↑).

The diameter of the knee joint in each group was significantly increased 1 day after the second sensitization (*p* < 0.01). One week following the second sensitization, the knee joint diameters of TP, TP-SLN, BS, DMSO and control group were (1.89 ± 0.07) cm (1.85 ± 0.06) cm, (1.83 ± 0.08) cm (2.09 ± 0.08) cm and (2.15 ± 0.08) cm, respectively. The diameter of the knee joint in the TP, TP-SLN and BS groups was significantly reduced compared to DMSO and control group (*p* < 0.01; Figure 4).

### 3.2 Ultrasonic Score of Knee Joint in Each Group

According to the scoring standards of Szkudlarek et al., Newman et al. and Disler et al., kappa values were 0.84, 0.81, and 0.78, respectively, demonstrating good consistency.

**FIGURE 5:**
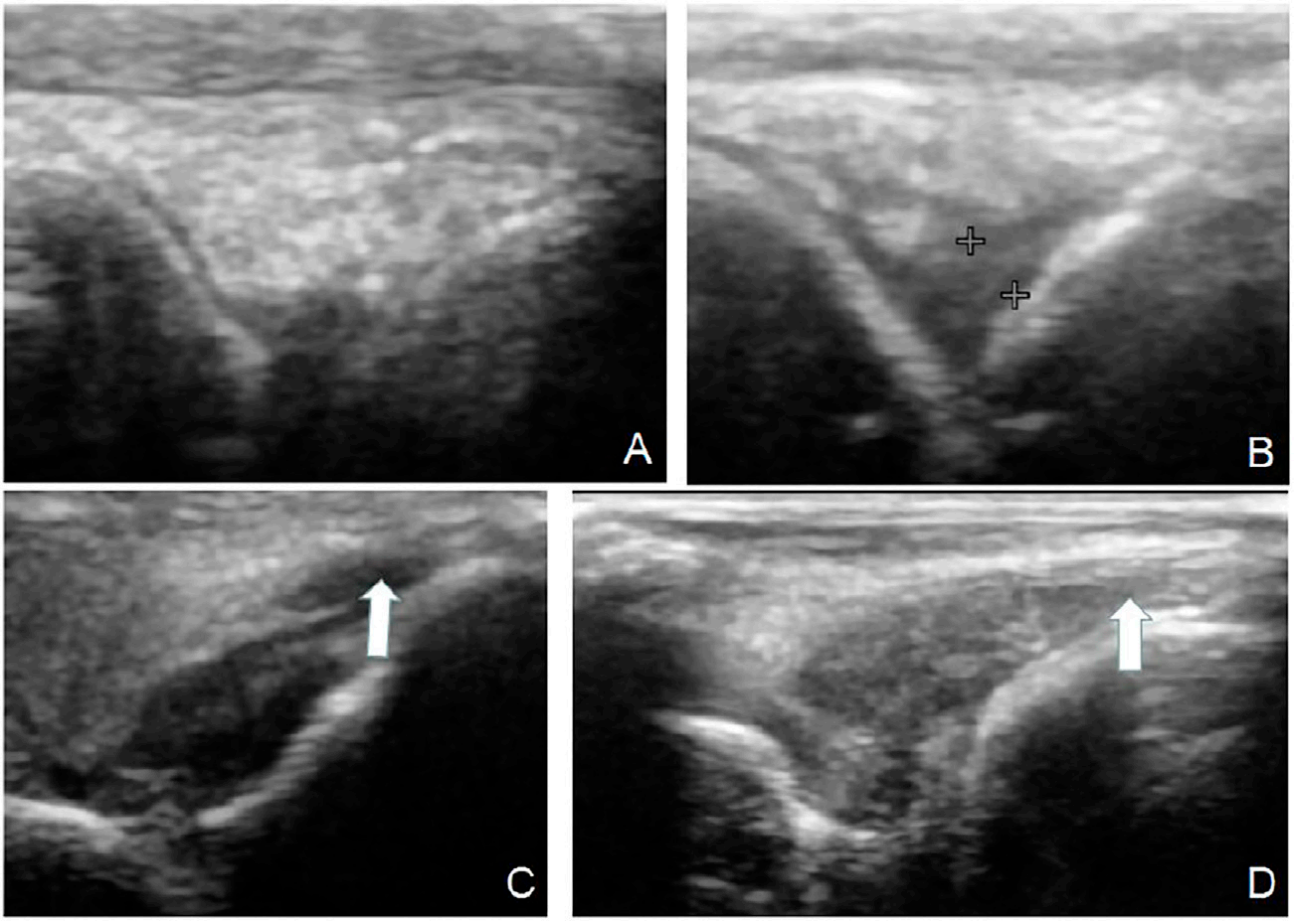
Ultrasonic evaluation of synovitis. Score 0: no obvious thickening of synovium. (TP-SLN group, **(A)**); score 1: mild synovitis can be seen (+ +), but it does not exceed the line of the highest point on the bone surface (TP group, **(B)**). Score 2: the line is higher than the highest point of bone surface, but not higher than the backbone (DMSO group, **(C)**). Score 3: over the line of the highest point of the bone surface and extending over one side of the shaft (control group, **(D)**).

As treatment progressed, the synovitis score in the control and DMSO groups increased gradually, but the synovitis of TP, BS and TP-SLN groups did not increase significantly. Some rabbit’s scores decreased. At the fifth week, the synovitis score in the control group and DMSO group was mainly 2 and 3, in the BS and TP-SLN groups the score was mainly 0 and 1, and in the TP group the score was mainly one and 2 (Table 1; Figure 5). The synovitis in the TP group was lower than in the control group (*p* < 0.05), while in the BS and TP-SLN groups the synovitis was lower than in the TP group (*p* < 0.05).

**TABLE 1:** The results of ultrasonography in the synovium of knee joint on the 35th day.

	0	1	2	3
TP	0	4	3	0
TP-SLN	3	4	0	0
BS	2	5	0	0
DMSO	0	1	3	2
Control	0	0	2	4

The scores of the BS, and TP-SLN, groups were lower than that of the TP, group (*p* < 0.05). The score of TP, group was lower than that of control group (*p* < 0.05).

**FIGURE 6:**
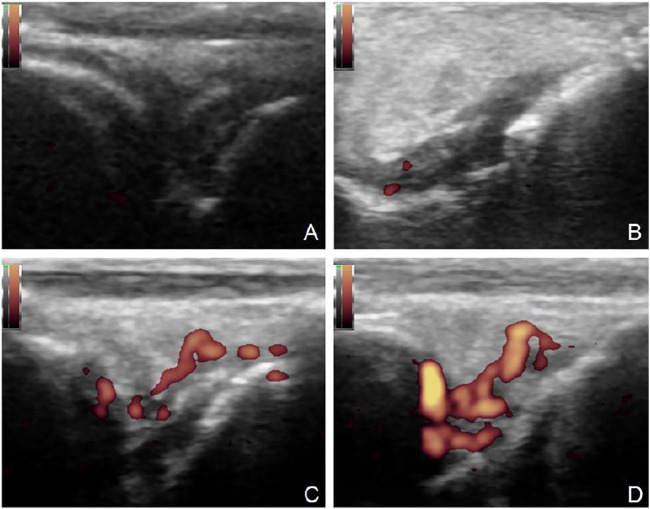
Power Doppler ultrasound of synovitis. Score 0: no color blood flow signal (TP-SLN group, **(A)**. Score 1: dot color blood flow signals (TP group, **(B)**). Score 2: short linear color blood flow signal with distribution less than 1/2 of synovial area (DMSO group, **(C)**). Score 3: reticular blood flow signals distributed more than 1/2 of synovial area (control group, **(D)**).

The synovial blood flow of the control group and DMSO group increased gradually. The synovial blood flow in the TP, BS and TP-SLN groups decreased gradually with the treatment. On the fifth week, the score for synovial blood flow in the TP, BS and TP-SLN groups was mainly 0, which was lower than that of the control group (*p* < 0.01). However, there was no significant difference among TP, BS and TP-SLN groups (*p* > 0.05; Table 2 and Figure 6).

**TABLE 2:** The results of ultrasonic evaluation of synovium blood flow of knee joint in the fifth week.

	0	1	2	3
TP	6	1	0	0
TP-SLN	5	2	0	0
BS	7	0	0	0
DMSO	0	4	1	1
Control	0	2	2	2

The scores of the TP, TP-SLN, and BS, groups were lower than that of control group (*p* < 0.05).

**FIGURE 7:**
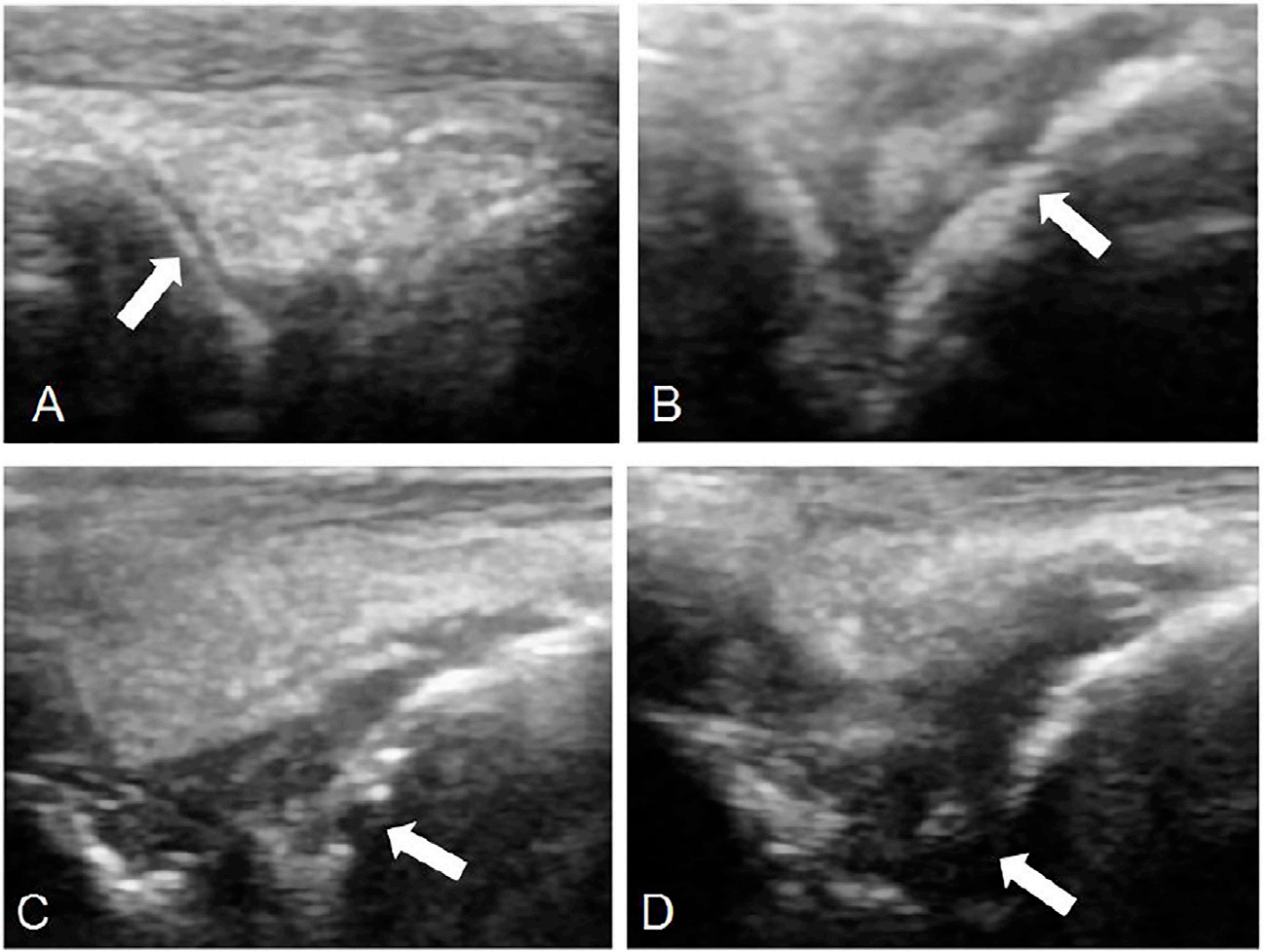
Observation of bone destruction by ultrasound. 0: No bone damage on the surface of bone (TP-SLN group, **(A)**). One point: The surface of bone is not smooth, but there is no bone destruction (TP group, **(B)**). Two points: Slight bone defect on bone surface (DMSO group, **(C)**). Three points: extensive bone destruction on bone surface (control group, **(D)**).

On the fifth week of ultrasound examination, the bone erosion scores in the TP, TP-SLN and BS groups were mainly 0 and 1, which were lower than that of the control group (*p* < 0.01). There was no statistically significant difference between TP, TP-SLN and BS groups (*p* > 0.05; Table 3 and Figure 7).

**TABLE 3:** The ultrasonic score of the destruction of the knee joint’s acoustic bone in the fifth week.

	0	1	2	3
TP	3	3	1	0
TP-SLN	5	2	0	0
BS	6	1	0	0
DMSO	0	2	3	1
Control	0	0	2	4

The scores of the TP, TP-SLN, and BS, groups were lower than that of control group (*p* < 0.05).

### 3.3 Pathological Score of Knee Joint

**FIGURE 8:**
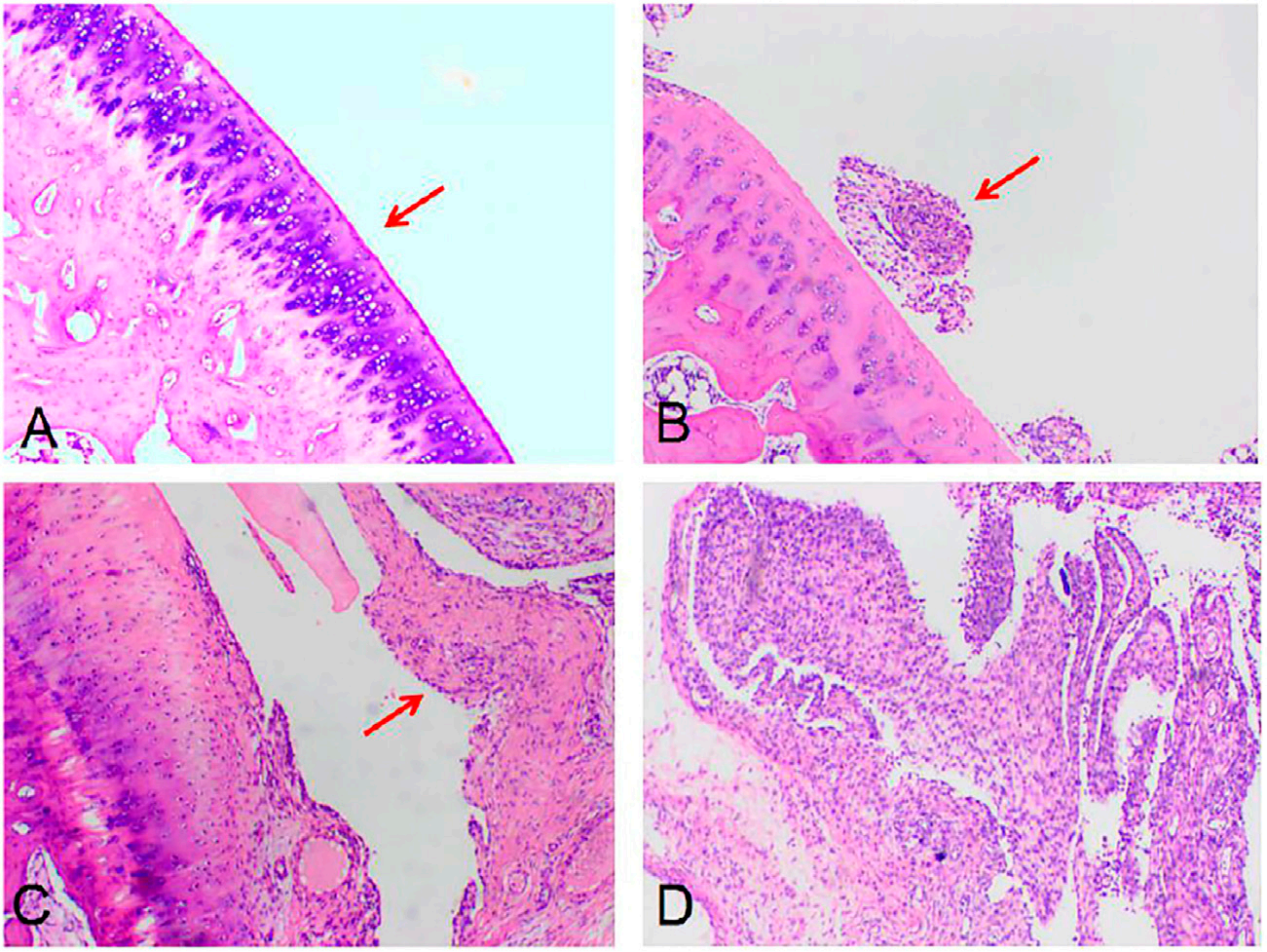
Pathological results of synovitis. The surface of bone and joint is smooth without synovium hyperplasia (TP-SLN and BS group, **(A)**). Mild synovial hyperplasia, a small number of inflammatory cells and synovial cells increased (TP group, **(B)**). Severe synovial hyperplasia with a large number of inflammatory cell infiltration and ulceration of lining layer (DMSO and control group, **(C)** and **(D)**) (red arrow, HE, 40×).

The synovial pathological score of the TP group was lower than that of the control group (*p* < 0.05), while in the TP-SLN and BS groups the score was lower than in the TP group (*p* < 0.05). There was no significant difference between the BS and TP-SLN groups (*p* > 0.05; Table 4 and Figure 8).

**TABLE 4:** Pathological score of knee synovium in each group.

	Normal	Mild	Severe
TP	2	5	0
TP-SLN	6	1	0
BS	7	0	0
DMSO	0	3	3
control	0	2	4

The scores of the BS, and TP-SLN, groups were lower than those of TP, group (*p* < 0.05). The score of the TP, group was lower than that of DMSO, and control groups (*p* < 0.05).

**FIGURE 9:**
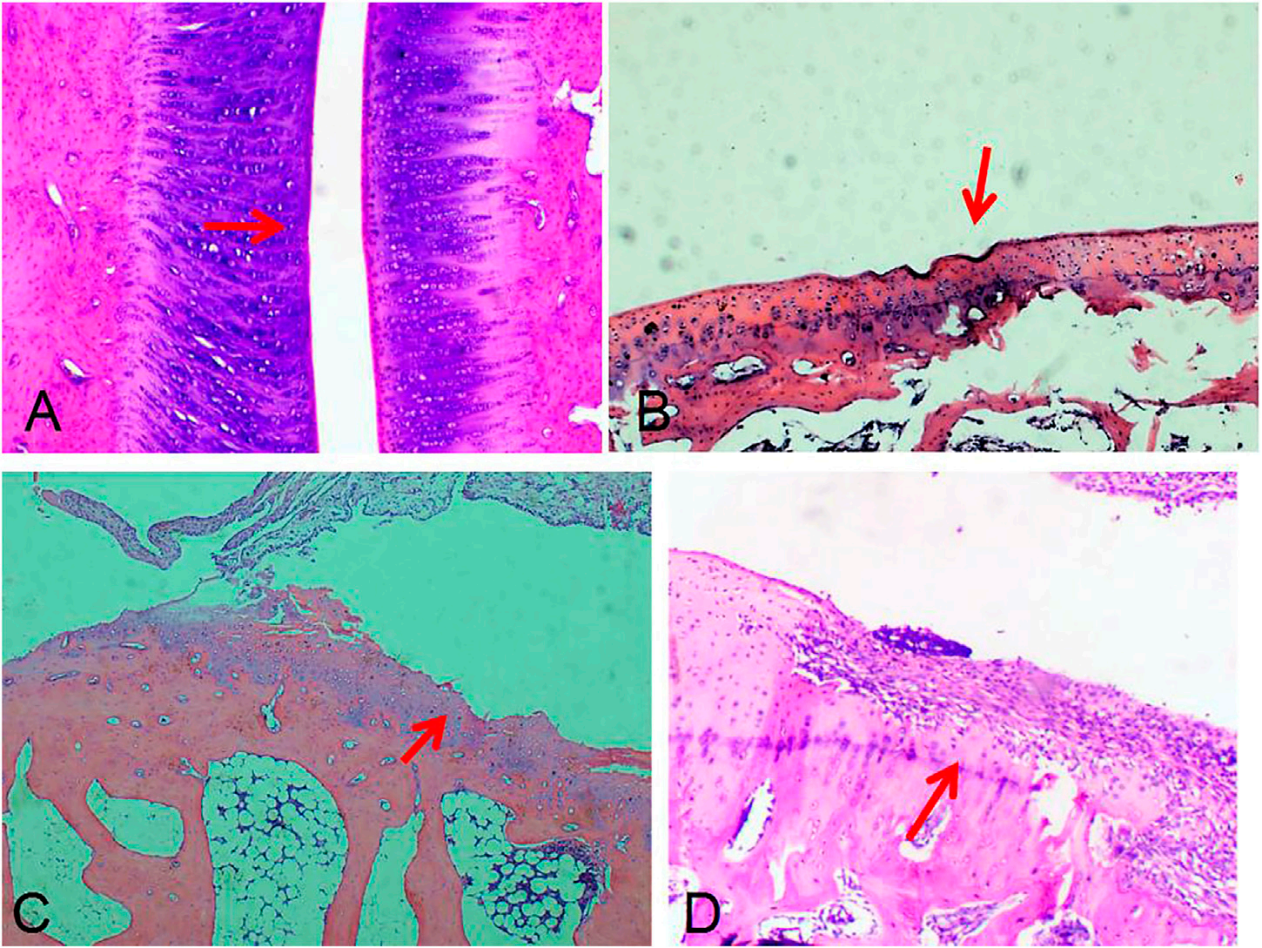
Pathological results of bone destruction. Score 0: the surface of bone was smooth and no bone damage was found (TP-SLN group, **(A)**). Score 1: the bone surface is not smooth (TP group, **(B)**). Score 2: slight bone defect on the bone surface (DMSO group, **(C)**). Score 3: extensive bone destruction on bone surface (control group, **(D)**). (red arrow, HE, 40×).

The pathological scores of bone erosion in the TP, TP-SLN and BS groups were all lower than in the DMSO and control groups (*p* < 0.05). There was no statistically significant difference among the TP, TP-SLN and BS groups (*p* > 0.05; Table 5 and Figure 9).

**TABLE 5:** Pathological results of bone destruction of the knee joint in each group.

	0	1	2	3
TP	5	2	0	0
TP-SLN	6	1	0	0
BS	6	1	0	0
DMSO	1	2	2	1
control	1	2	1	2

The scores of the TP, BS, and TP-SLN, groups were lower than that of the DMSO, group.

The ultrasonic and pathological scores of synovitis and bone erosion in 33 rabbits were analyzed. A significant correlation was found between the two scoring methods in evaluating synovitis and bone erosion of knee joint (r = 0.832 and 0.859, respectively, *p* < 0.001).

## 4 Discussion

The AIA model in rabbits can simulate human RA disease process. In our study, 24 h after intra-articular injection of ovalbumin, the knee joints of all rabbits were significantly swollen (*p* < 0.05). The success rate of the AIA model was 100%. One week after intra-articular injection of TP, TP-SLN and BS, the swelling of the joints was significantly reduced. One month of continuous treatment by injecting TP into the articular cavity did have an effect on inhibiting synovitis and bone erosion (better than DMSO, *p* < 0.05), but the effect was not as good as treatment with TP-SLN and BS (*p* < 0.05).

The mechanism of TP in the treatment of RA has been widely studied. The main mechanism of TP is to regulate miR-155 to inhibit the immune response of monocytes (Li et al., 2021) and the signal transduction of myeloid cell trigger receptor-1 (TREM-1) (Fan et al., 2016), so as to control inflammation. TP has been used in the treatment of RA, but most of the patients have been treated by oral or intravenous injection. There is no report of direct intra-articular administration. In this study, the AIA arthritis model was established and TP-SLN was made. The rabbits were injected with TP-SLN in the knee joint and a therapeutic effect was observed. The results of this study showed that the anti-inflammatory effect of TP loaded with SLN was higher than that of TP alone.

TP-SLN did not change the physical and chemical properties of TP. The reason why TP-SLN has higher anti-inflammatory effect than TP is the change of its pharmacokinetics. At present, two main mechanisms have been described. Chetoni et al. (2016) found that the concentration of liposome-encapsulated tobramycin (tobra-SLN) in eye tissue is higher than in a non-encapsulated control group. Compared with traditional medicine, it can also penetrate into the retina and tobra-SLN has a higher bactericidal effect on intracellular *Pseudomonas aeruginosa*. In the study of the pharmacokinetics of tobra-SLN, it was found that in the nanoparticles tobramycin was concentrated in lipids and did not interact with stearic acid. It can slowly dissolve into the medium, resulting in constant release of the drug, which can increase the action time of the drug into the target tissue to enhance the efficacy. Ramalingam and Ko (2015) found that curcumin encapsulated by SLN improves oral bioavailability because SLN can protect curcumin from degradation by enzymes in gastrointestinal tract, and it can stay in the gastrointestinal tract for a long time. Based on the physicochemical properties of TP, the possible reasons that TP-SLN increases the anti-inflammatory effect of TP are as follows. First, water-soluble substances are easier to pass through the synovium. However, TP is difficult to dissolve in water, does not easily pass through synovial fluid, and may be easily swallowed by macrophages, which may affect the efficacy. The particle size of SLN is about 50–1,000 nm, which is one of the most effective methods to improve the bioavailability of water-soluble substances (Papakostas et al., 2011). Secondly, there are hyaluronidase and other enzymes in the joint cavity, and TP may be easily degraded by enzymes after direct injection, while SLN can play a protective role. Third, the metabolism in the joint cavity is relatively fast, and the foreign material will be removed quickly. TP-SLN may stay in the joint cavity for a longer time and release TP more slowly and stably. However, the specific mechanism needs further study.

Ultrasound examination plays a very important role in the assessment of rheumatoid arthritis. Compared with serological examination, it can effectively evaluate whether rheumatoid arthritis is in an active stage and clinical symptoms of patients by observing the blood flow signal richness in the synovium with power Doppler imaging (PDI) (Alekseeva et al., 2015). In animal experiments, it was found that the earliest high-frequency ultrasound detection of synovium thickening was 1 day after the injection of ovalbumin into the articular cavity and the latest was 7 days. The significant increase of VEGF and TNF-α in serum appeared on day 14 at the earliest and day 42 at the latest. Therefore, the sensitivity of ultrasonography in the diagnosis of early rheumatoid arthritis is higher than that of serology, and it can also effectively evaluate the active arthritis.

Our results show that the pathological score of synovitis has a good correlation with the ultrasound score. The positive rate of ultrasound score was even higher than that of pathological score. The reasons may be as follows. First, hyperemia and edema are two important pathological changes of inflammation, and ultrasound can identify the synovial thickening and the increase of blood flow signal caused by hyperemia and edema. However, pathological examinations were carried out after the animals were sacrificed. After a series of processes such as decalcification, dehydration and paraffin embedding, the extent of congestion and edema will be lower than that of living animals. In the process of making pathological specimens, we also found that the tissue mass after decalcification and dehydration was obviously atrophic. Secondly, the pathological section only selected several sections of the rabbit knee joint, but the ultrasound allows a more comprehensive observation. Especially in the case of a knee joint with mild synovitis, ultrasound will detect the inflammation, but the pathological section may not be selected in the synovitis area. Thirdly, the pathological score is related to the infiltration degree of inflammatory cells, while the ultrasonic score is only to observe the changes of synovium morphology. In the TP, TP-SLN and BS groups, the inflammation may be improved after 1 week of treatment, so the pathological score will be reduced.

In summary, our study found a high correlation between ultrasound and pathology scores of synovitis and bone erosion. Ultrasound can provide useful assessment of synovitis in early arthritis. We also demonstrated that early treatment with TP-SLN is an effective strategy for suppressing joint inflammation and preventing subsequent joint damage in RA and that the anti-inflammatory effect of TP-SLN is greater than that of TP.

## 5 Conclusion

The therapeutic effect of TP-SLN on arthritis is better than that of TP, but there is no difference between BS and TP-SLN. Therefore, TP-SLN may be used as an alternative to BS in the treatment of rheumatoid arthritis in the future. The ultrasonic and pathological scores showed significant correlation in synovitis and bone erosion. Ultrasound can provide a useful assessment of synovitis in early arthritis.

## Data Availability

The original contributions presented in the study are included in the article/Supplementary Material, further inquiries can be directed to the corresponding author.
